# Clinical and histologic tissue responses of skin incisions closed with glycomer 631 and lactomer 9–1 using a subcuticular technique in pigs

**DOI:** 10.1186/s12917-020-02290-x

**Published:** 2020-02-24

**Authors:** Tanja Plavec, Tanja Švara, Irena Zdovc, Mitja Gombač, Marija Damjanovska, Tatjana Stopar Pintarič, Erika Cvetko, Alenka Seliškar

**Affiliations:** 1 Small Animal Clinic, Veterinary Faculty, University of Ljubljana, Gerbičeva 60, 1000 Ljubljana, Slovenia; 2 Institute of Pathology, Wild Animals, Fish and Bees, Veterinary Faculty, University of Ljubljana, Gerbičeva 60, 1000 Ljubljana, Slovenia; 3 Institute of Microbiology and Parasitology, Veterinary Faculty, University of Ljubljana, Gerbičeva 60, 1000 Ljubljana, Slovenia; 4 Clinical Department of Anaesthesiology and Intensive Therapy, University Medical Centre Ljubljana, Zaloška 7, 1000 Ljubljana, Slovenia; 5 Institute of Anatomy, Faculty of Medicine, University of Ljubljana, Vrazov trg 2, 1000 Ljubljana, Slovenia

**Keywords:** Pig, Absorbable suture material, Glycomer 631, Lactomer 9–1, Tissue reaction, *Trueperella pyogenes*

## Abstract

**Background:**

Glycomer 631 and lactomer 9–1 are absorbable suture materials indicated for soft tissue approximation in non-infected wounds. Pigs are often used as surgical models in translational research; however, reports of tissue reactions to both suture materials in pigs are limited. The aim of this study was to assess clinical and histologic responses of skin incisions closed with a subcuticular technique using glycomer 631 and lactomer 9–1 in pigs.

**Results:**

Skin incisions on 17 pigs were closed with glycomer 631 and lactomer 9–1, and a clinical reactive score (CRS) including erythema, swelling, discharge and dehiscence was calculated on postoperative days 7 and 14. Subcuticular tissue reaction was assessed histologically on postoperative day 14 (the presence of extravascular neutrophils, macrophages, multinucleated giant cells, lymphocytes, fibroblasts, bacterial colonies and the overall severity of the inflammatory response to the suture material), and the cumulative score of the variables was calculated as an aggregate tissue irritation score (ATIS). Tissue samples were examined for suture extrusion and evaluated microbiologically. The clinical reactive score did not differ between the suture materials. Only one ATIS variable, namely the overall severity of the inflammatory response, was lower (*p* = 0.029) when glycomer 631 was used. Suture extrusion was found in 10/17 of the incisions closed by glycomer 631 and in 7/13 of the incisions closed by lactomer 9–1. *Trueperella pyogenes* was isolated from the skin and from the area of tissue reaction in six pigs.

**Conclusions:**

No difference in CRS between the suture materials was observed, and thus both materials may be used for the subcuticular technique in pigs. Glycomer 631 induced less tissue reaction only in terms of the overall severity of the inflammatory response. Suture extrusion was observed in more than 50% of incisions regardless of the suture material, possibly due to a large amount of suture material in the wound. *Trueperella pyogenes* was the only pathogen isolated from the tissue surrounding the suture material.

## Background

Primary wound closure is accomplished through subcutaneous tissue apposition followed by either cutaneous or buried subcuticular suture placement [1].

Cutaneous sutures with non-absorbable suture materials are easy to perform and provide excellent apposition of wound edges. The disadvantages are the need for removal, bacterial migration along suture tracts, scarring and skin irritation leading to self-mutilation [2].

The buried continuous subcuticular suture pattern with absorbable suture material provides exceptional skin edge apposition, which promotes rapid epithelization in dogs [3] and does not increase the degree of inflammation in noncontaminated wounds in rats [4]. Swelling and erythema may occur initially because of increased intraoperative tissue handling when compared with cutaneous sutures. Further, scar formation is decreased, and better cosmetic appearance of the skin closure was noted in dogs 10 to 14 days after ovariohysterectomy when compared to a single interrupted suture pattern [5]. In addition, there is no need for suture removal [6], and the risk for self-induced trauma is reduced due to the absence of protruding material for the animal to scratch or lick [3].

Both monofilament glycomer 631 (Biosyn, Covidien, USA) and braided lactomer 9–1 (Polysorb, Covidien, USA) are absorbable suture materials indicated for soft tissue approximation in non-infected wounds. Glycomer 631 is composed of glycolide, dioxanone and trimethylene carbonate. Very low tissue reaction was observed when skin incisions were closed with glycomer 631 with a buried vertical intradermal suture pattern in rats [7]. However, when glycomer 631 was compared with polyglycolic acid, polypropylene and tissue adhesive for the subcuticular closure technique in rats, tissue fibrosis and inflammatory cell infiltration were more prominent with glycomer 631 and polypropylene, and foreign body giant cells were present in the glycomer 631 group [8]. In contrast, lactomer 9–1 is a synthetic polyester composed of glycolide and lactide. In rats it caused marked inflammation around the suture 10 days after implantation into the gluteal muscles. The reaction dissipated for a period of 3 months after implantation, after which no sign of tissue response was detected [9]. In miniature pigs, subcuticular skin closure with lactomer 9–1 resulted in a 19% cumulative incidence of suture extrusion observed macroscopically, with the volume of suture material in the wound being a critical determinant of suture extrusion [10].

Glycomer 631 and lactomer 9–1 are materials frequently used in small animal practice, but reports of clinical and histological tissue reactions to these two materials in pigs are limited to only one study on lactomer 9–1 [10]. Pigs are often used as surgical models in translational research; therefore, the purpose of this study was to assess the clinical and histologic responses of skin incisions to subcuticular closure with glycomer 631 or lactomer 9–1 in this animal species. As studies in dogs have reported fewer clinical and histologically proven reactions to monofilament versus multifilament sutures [3, 11], we hypothesised that in pigs monofilament glycomer 631 would induce less tissue reaction than braided lactomer 9–1 suture material.

## Results

### Clinical observations

All pigs recovered uneventfully after surgery. No signs of discomfort or pain were observed in the post-operative period, and all pigs were walking, eating and drinking normally six to 8 h after surgery. No additional analgesia with butorphanol was required beyond 12 h after the end of anaesthesia.

**Fig. 1:**
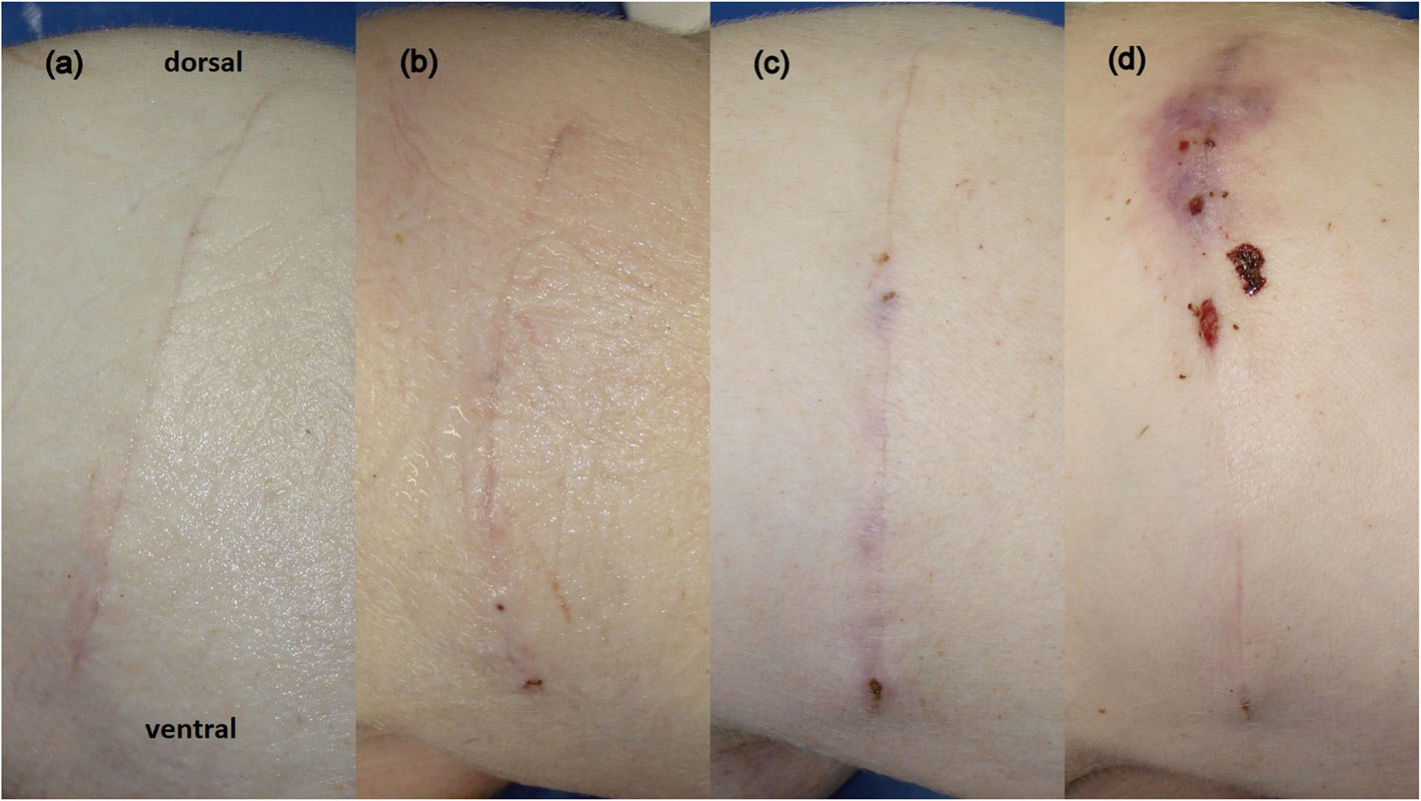
Macroscopic appearance of skin incisions on day 14 postoperatively. **a** CRS 0, **b** CRS 0 dorsally and 2 ventrally, **c** CRS 0 dorsally and 3 ventrally, and **d** CRS 8 dorsally and 2 ventrally. CRS = clinical reactive score

During surgery, both suture materials were handled easily, and a successful wound approximation was achieved. The macroscopic appearance of skin incisions on day 7 and 14 postoperatively (Fig. 1) did not differ between the suture materials with respect to CRS (Table 1).

**Table 1:** Clinical reactive score (CRS) 7 and 14 days after subcuticular skin closure by use of glycomer 631 and lactomer 9–1 in pigs; values are expressed as median (minimum, maximum). The maximum possible CRS is 12. No difference between the suture materials was observed

Postoperative day	Glycomer 631	Lactomer 9–1	*p-*value
7	1 (0, 8)	0 (0, 8)	0.120
14	1 (0, 7)	0 (0, 8)	0.614

### Histopathologic evaluation

**Fig. 2:**
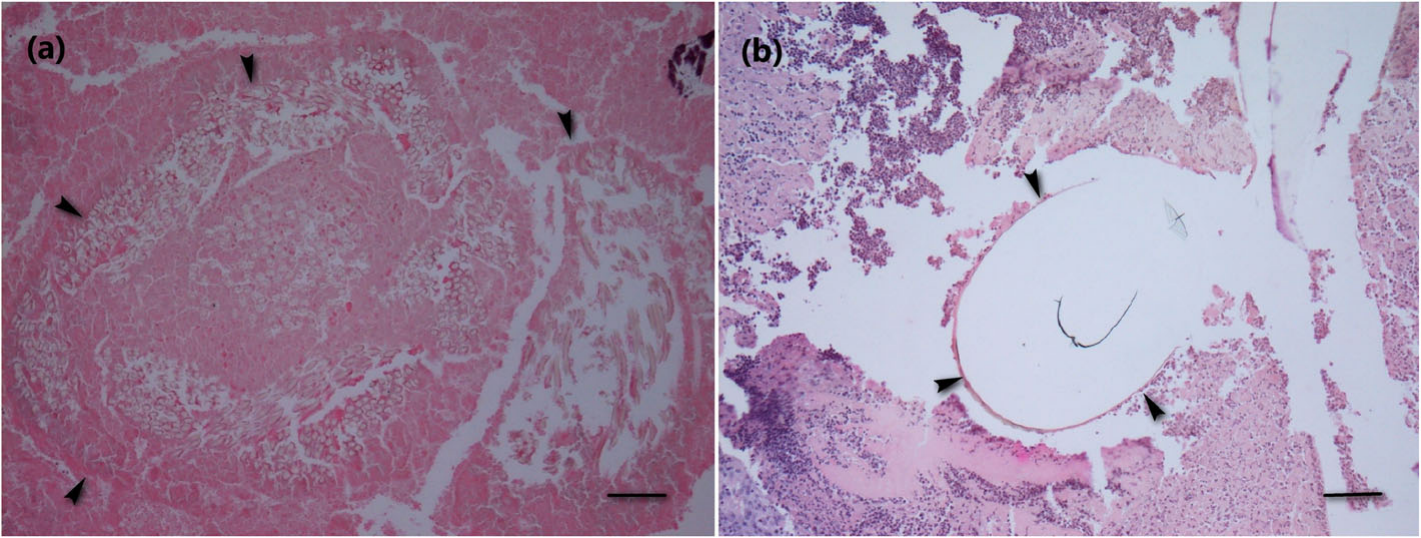
**a** Abscess with remnants of lactomer 9–1 (arrowheads). Bar = 100 μm. **b** Abscess with remnants of glycomer 631 (arrowheads). Bar = 100 μm

The dermal tissue reaction in all samples included a variably wide zone of granulation tissue rich with collagen and was infiltrated predominantly with macrophages and less numerous lymphocytes, which extended from the epidermis into the subcutis. Suture material was typically found within abscesses, granulomas and pyogranulomas (Fig. 2). Glycomer 631 was found in all samples. Lactomer 9–1 was detected only in 13 of 17 samples, for which the assessment was carried out and the results of the ATIS presented. In abscesses, the suture material was surrounded with liquefactive necrosis, numerous neutrophils and less numerous macrophages encircled with a rim of fibrous connective tissue capsule. Granulomas were composed of suture material surrounded with macrophages, less numerous Langhans multinucleated giant cells and lymphocytes, and encircled with a rim of fibrous connective tissue capsule. In the surrounding dermis, single multinucleated giant cells or small groups of these cells forming small granulomas were observed. The remains of suture material were noticed even in the cytoplasm of some giant cells. In pyogranulomas, the suture material was encircled by a mixture of neutrophils, macrophages, Langhans multinucleated giant cells and lymphocytes. The difference between glycomer 631 and lactomer 9–1 was observed in only one ATIS variable. The overall severity of the inflammatory response was lower (*p* = 0.029) with glycomer 631 compared to lactomer 9–1 (Table 2).

**Table 2:** Aggregate tissue reactive score (ATIS) 14 days after subcuticular skin closure by use of glycomer 631 and lactomer 9–1 in pigs; values are expressed as median (minimum, maximum). Significant difference was observed only in overall severity of inflammatory response to SM

	Glycomer 631	Lactomer 9–1	Maximum possible score	*p-*value
Extravascular neutrophils	−12 (0, − 24)	− 12 (0, − 30)	−30	0.340
Macrophages	−1 (− 1, − 3)	− 2 (− 1, − 2)	−5	0.079
Multinucleated giant cells	0 (0, − 2)	−2 (0, − 2)	− 10	0.439
Lymphocytes	−2 (0, − 6)	−4 (− 2, − 8)	−10	0.140
Fibroblasts	−2 (−1, − 4)	−2 (− 1, − 5)	−5	0.171
Overall severity of inflammatory response to SM^a^	−3 (− 3, − 9)	−6 (− 3, − 15)	−15	0.029
Bacterial colonies	0 (0, −9)	0 (0, − 9)	−9	0.589
ATIS	−20 (−7, − 54)	− 25 (− 10, − 69)	− 84	0.111

*SM* suture material

^a^Significant difference between glycomer 631 and lactomer 9–1

**Fig. 3:**
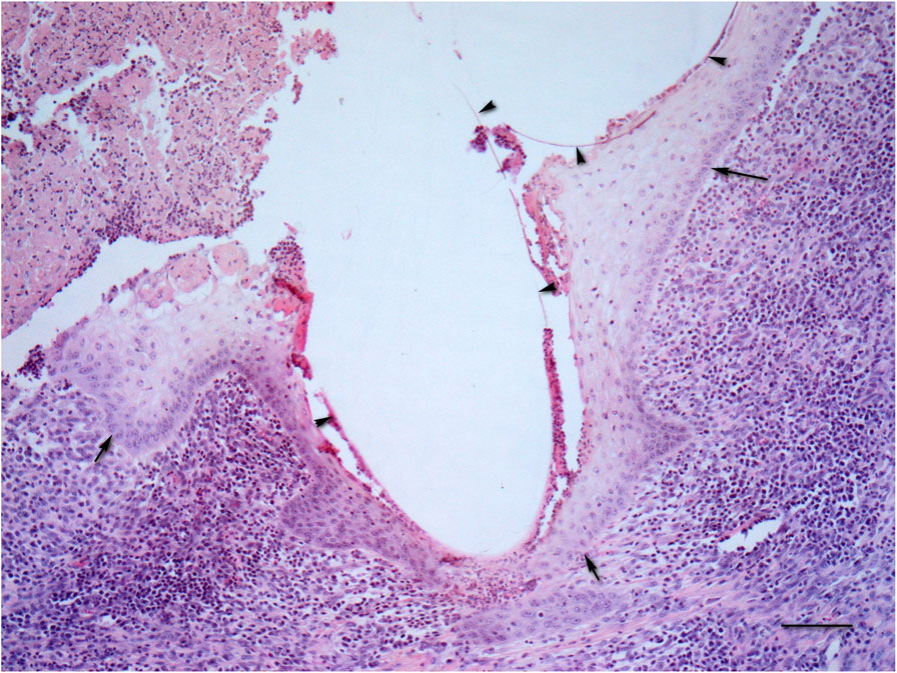
Skin incision closed by glycomer 631. Suture material (arrowheads) partially encircled with a rim of stratified squamous epithelium (arrows). Bar = 100 μm

**Fig. 4:**
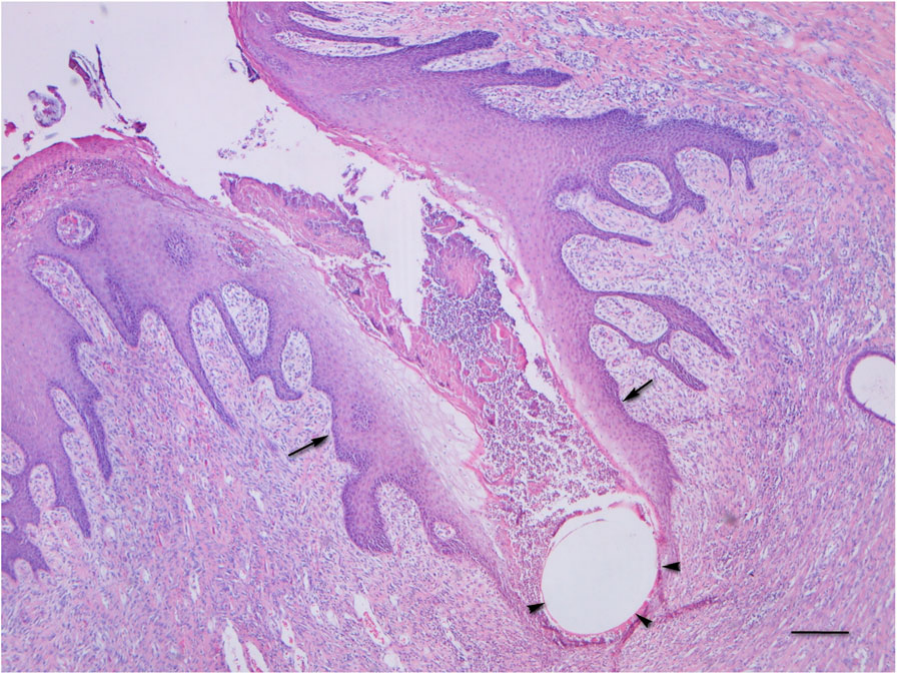
Skin incision closed by glycomer 631. Suture material (arrowheads) in the tract covered with stratified squamous epithelium opened to the skin’s surface (arrows). Bar = 100 μm

Suture material in the dermis was frequently partially encircled with a rim of stratified squamous epithelium (Fig. 3) or found inside a tract covered with stratified squamous epithelium opened to the skin’s surface (Fig. 4). The latter was found only once with each suture material, while in the remaining samples suture material was not in contact with the skin surface. The suture extrusion was found in 10/17 samples closed by glycomer 631 and in 7/13 samples closed by lactomer 9–1; the difference between suture materials was not significant (*p* = 1.0). Histopathologic evaluation revealed almost complete epithelialization in both groups. The area of the incision was covered with mildly or moderately hyperplastic epidermis in 16/17 samples closed by glycomer 631 and in 11/13 samples closed by lactomer 9–1; the difference between suture materials was not significant (*p* = 0.565). Single small pustules were found in the epithelialization area in 3/17 samples sutured with glycomer 631 and in 3/13 samples sutured with lactomer 9–1; the difference between suture materials was also not significant (*p* = 1.0).

### Microbiological examination

Bacterial growth was observed from the skin and the area of tissue reaction of six pigs, and *Trueperella pyogenes* was the only pathogen isolated. Bacterial cells that morphologically correspond to *Trueperella pyogenes* were also found histologically in the area of tissue reaction of the same pigs (three pigs in which glycomer 631 was used and thee pigs in which lactomer 9–1 was used). Bacterial culture was not performed to answer that question.

## Discussion

Macroscopic appearance of the skin incisions or CRS on day 7 and 14 postoperatively did not differ between the suture materials used in this study. Similarly, Kirpensteijn et al. [3] did not observe any changes in macroscopic appearance when monofilament poliglecaprone 25 was compared with braided polyglactin 910 for closure of canine skin incisions at 7, 14 and 28 days postoperatively. However, significantly less redness and swelling were detected the day after the surgery when the monofilament poliglecaprone 25 suture was used [3]. This difference was attributed to the pronounced tissue reactivity and increased friction in the tissues related to the multifilament structure of polyglactin 910 [11]. Non-absorbable monofilament polypropylene used for the subcuticular pattern in cats resulted in even less reaction when compared to the absorbable monofilament copolymer of glycolide, ε-caprolacton and trimethylene-carbonate material [12], probably due to the absence of a resorption-associated reaction of the tissue to the polypropylene suture and absence of knots in the tissue, since the suture was secured through a fixation clip on the skin surface. Another factor could be the amount of tissue handling necessary for suture placement. It has been shown that wounds closed by the subcuticular pattern using poliglecaprone 25 showed significantly more swelling and erythema on the first postoperative day compared to the simple interrupted pattern with either poliglecaprone 25 or polypropylene sutures [5].

In summary, macroscopically detectable tissue reaction due to surgical manipulation and greater friction of multifilament suture material can be seen on the first postoperative day [3, 5, 11], subsiding until day 7 postoperatively [3, 5]. This is in agreement with our observations on day 7 and 14 postoperatively when the CRS of both suture materials was low and no differences among them were confirmed macroscopically.

Histopathologic evaluation of wound healing is more sensitive and precise compared to macroscopic assessment; however, none of the methods for histopathologic evaluation are universally accepted and usually compare suture materials by the infiltration of predominant inflammatory cells [3, 4, 7, 13]. The suture material in our study was surrounded with liquefactive necrosis, numerous neutrophils and less numerous macrophages, encircled with a rim of fibrous connective tissue capsule, but there was no difference in the presence of neutrophils in the proximity of both suture materials. The only difference between lactomer 9–1 and glycomer 631 regarding histopathologic evaluation on day 14 was the lower overall severity of the inflammatory response when glycomer 631 was used. Molea et al. [7] also reported neutrophil reaction/acute inflammatory reaction to glycomer 631 in rats. In another study, predominantly neutrophilic infiltrate was observed in incisions sutured with polyglactin 910 in a continuous subcuticular pattern and with metal staples as well [13]. Further, Kirpensteijn et al. [3] observed a lower mean tissue reaction score with polyglecaprone 25 compared with polyglactin 910 on day 7 in dogs, but this difference was not evident on day 14 after skin surgery. Unfortunately, we do not have data for day 7 because biopsies were only taken on day 14. The observed differences between the suture materials in Kirpensteijn’s study [3] and those in our study might be due to the multifilament nature of lactomer 9–1 and polyglactin 910 resulting in increased tissue trauma around the suture path [1, 3]. Also, both sutures might incur increased inflammation due to possible transient infiltration of bacteria in their multifilament structure [14]; however, bacteria were isolated only in the present study.

The proliferation of the squamous stratified epithelium around suture material and/or its expulsion through the skin, interpreted as a suture extrusion [10], occurs in young growing pigs when suture material with a longer resorption time is used [15].

Suture extrusion occurred in more than 50% of incisions sutured with glycomer 631 or lactomer 9–1 in our study. This finding indicates that suture extrusion in pigs may occur even earlier than that stated for humans, that is at three to 8 weeks [16]. The skin of both species is comparable and, unlike that in rodents and carnivores, is tightly attached to the subcutaneous tissues. However, it is also thicker and less vascular than human skin, which makes direct comparison between species difficult [15, 17–19]. Further, skin on the back is thicker than abdominal skin, and perfusion measurements of the gluteal/thigh region [17], which also lack the panniculus carnosus [20], showed much lower blood flow when compared to the ventral abdominal region [17]. Jonsson et al. [21] have proven that collagen deposition and wound healing were directly and significantly proportional to wound oxygen tension and perfusion, hence the selection of a less vascular region in our study may have had a detrimental effect on wound healing and contributed to the unfavourable results when compared with studies where incisions were performed in the well vascularised ventral abdominal region [3, 5, 7, 12, 13]. Supporting this finding, Drake at al. [10] reported that the incidence of extrusion at the joint was twice as great as that on the abdomen with the larger 5-throw knot. The same study compared two multifilament sutures, where polyglactin 910 had a higher cumulative incidence of suture extrusion than lactomer 9–1 (31% versus 19% at 5 weeks) in juvenile Hanford minipigs, the rate depended on the volume of suture material in the wound. The 5-throw surgeon’s knot with lactomer 9–1 had a higher incidence of suture extrusion than knots with less throws but did not exceed 17 and 30% at two and 5 weeks, respectively.

The exact reason for the higher incidence of suture extrusion in our study is not completely understood. As previously suggested, suture extrusion may occur in tissues with poor blood supply or may be related to the degree of tissue reactivity of the suture material, more superficial dermal placement [22], the mechanical motion of the knots on extremities or the volume of suture material in the wound [10]. All of these aetiologies could explain the suture extrusion in our study; however, given that we used 6-throw knots, the volume of suture material seems the most plausible explanation.

*Trueperella pyogenes* is a Gram-positive, rod-shaped, opportunistic, ubiquitous bacterium, which may be a part of the normal skin, oropharyngeal, urogenital and gastrointestinal flora of animals [23]. It was isolated from the skin and tissue around the suture material of six pigs in our study. In pigs it usually causes abscesses [23, 24], which were noted in the proximity of suture material in this study. The fact that *Trueperella pyogenes* was isolated from both the skin and tissue surrounding the sutures in the same pigs suggests that the infection was spread from the skin during or soon after surgery despite careful aseptic preparation of the skin, where the contact time of the antiseptic well exceeded the exposure time recommended by the manufacturer. However, surgical preparation of the skin does not eliminate bacteria from the skin but only reduces the quantity [25]. Further, epithelial cells bridge the minor gap in sutured wounds in 48 h [26], and during this period contamination of suture material is possible. Also, since pigs do not live in a sterile environment, the presence of bacteria on the skin and in incisions is expected even if pens are kept as clean as possible.

Fick et al. [13] observed purulent exudate along one incision closed with polyglactin 910 and with metal staples, respectively, and they also attributed it to environmental contamination given the absence of overt evidence of clinical infection. Even though braided suture materials tend to have a greater capillarity effect [14], no difference was observed between glycomer 631 and lactomer 9–1 regarding the bacterial colonization of tissue surrounding the suture material in our study. A possible explanation is the caprolactone/glycolide copolymer and calcium stearoyl lactylate coating on the lactomer 9–1, which is designed to facilitate knot formation [14] and acts as an antimicrobial agent [27].

*Trueperella pyogenes* was isolated from tissue surrounding the suture material in 6 of 17 pigs. No antibiotic sensitivity was determined in our study; however, in vitro antimicrobial sensitivity of *Trueperella pyogenes* isolated from domestic animals demonstrated good sensitivity to amoxicillin (94.6%) [24]. The reasons for the antibiotic ineffectiveness in our study could be (1) the fact that *Trueperella* forms abscesses where the antibiotic is more difficult to penetrate [28]; (2) low bioavailability of orally administered amoxicillin and clavulanic acid in pigs, which was found to be 22.8% for amoxicillin and 44.7% for clavulanic acid [29]; and (3) inappropriate dosing of the antibiotic since amoxicillin has a short half-life when given orally and should be given twice a day [30].

Our study has several limitations. First, the placement of suture material was not randomised. Lactomer 9–1 was used only in the dorsal part of the wound and glycomer 631 only in the ventral part of the wound. Since blood flow in different body regions varies [17], the blood vessel density in the dorsal and ventral parts of the wound may differ and could impact tissue healing. Also, only the left thigh was used for incisions, and the vascular supply of the skin might be different between the two limbs. Second, only 13 samples of the tissue sutured with lactomer 9–1 were analysed because in four of them the suture material could not be visualised at histopathological examination. Third, antibiotic prophylaxis with amoxicillin-clavulanic acid was extended to day 10 after surgery because of the requirements of the primary study, and, although amoxicillin-clavulanic acid should be given orally twice a day to be effective [30], it may have masked the microbiological analysis of tissue samples. It is not typical to use an antibiotic after clean surgical procedures [31], yet we decided in its favour due to the stable environment, inability for postoperative surgical dressing of incisions on the lateral aspect of the thigh and interaction of the pigs. The use of antibiotics in farm animals is restricted to keep drug-resistant bacteria out of food production; however, in experimental pigs that are not intended for food production, the exceptional use of antimicrobial drugs is allowed according to national and European legislation.

## Conclusions

There was no difference between the suture materials with respect to the CRS and thus both materials may be used for subcuticular sutures in pigs. Monofilament glycomer 631 induced less tissue reaction only in terms of the overall severity of the inflammatory response. Suture extrusion was observed in more than 50% of incisions regardless of the suture material, and *Trueperella pyogenes* was the only pathogen isolated from the tissue surrounding the suture material. Future studies could include comparisons of knot and suture size to determine the cause of suture extrusion in pigs.

## Methods

This study was conducted in accordance with ARRIVE (Animal Research: Reporting of in Vivo Experiments) guidelines. All procedures involving the experimental use of animals were approved by the National Ethics Committee and National Veterinary Administration (licenses No U34401–28/2013/7, approval date 14.10.2013 and No U34401–28/2013/17, approval date 29.7.2014). All procedures complied with the relevant national and European legislation.

### Animals and housing

Fourteen female and three castrated male pigs from a Landrace and Large White cross, aged eight to 9 weeks and weighing approximately 20 kg (weight estimated with a weight measuring tape for pigs) were used in the study. Following the 3Rs principles (replacement, reduction and refinement), we performed this study in pigs that were primarily included in a neurological study in which the sciatic nerve was surgically exposed. The pigs were reared according to the Council directive for minimum standards for the protection of pigs (2008/120/EC). Three days before they were anaesthetised, they were procured from a certified commercial farm (Globočnik Aleš, Voglje, 4208 Šenčur, Slovenia; KMG-MID SI100205065) free of classical and African swine fever and Aujeszky’s disease. The pigs were vaccinated against *Mycoplasma hyopneumoniae* and porcine circovirus type 2. The pigs were clinically examined and housed in indoor straw-bedded pens of appropriate size (minimum 2 m^2^ per animal) in groups of four or five animals originating from the same nest. They were exposed to a natural light/dark cycle and kept at a room temperature of 20 to 23 °C. They were fed commercial pig feed for growers twice a day and had unlimited access to tap water from nipple waterers. Food was withheld from them 12 h before anaesthesia.

### Anaesthesia protocol

Pigs were premedicated with midazolam 0.5 mg/kg, butorphanol 0.5 mg/kg and ketamine 10 mg/kg mixed together and given intramuscularly approximately 5 cm behind and on the level of the base of the ear. An intravenous catheter was placed into an auricular vein, and anaesthesia was induced with propofol, titrated to effect. After endotracheal intubation, anaesthesia was maintained with isoflurane at 1.5 to 2% (vapour setting) in a mixture of oxygen (1 L/min) and air (1 L/min) delivered through a circle breathing system. The pigs breathed spontaneously during anaesthesia.

The pigs were placed in right lateral recumbency on an electrically heated surgery table. Arterial oxygen saturation (SpO_2_), end-tidal CO_2_ concentration, oesophageal temperature, ECG and blood pressure (non-invasively) were monitored. Hartmann’s solution was infused intravenously during anaesthesia at a rate of 10 mL/kg/h. The pigs recovered from anaesthesia in a warmed, straw-bedded pen. The endotracheal tube was removed when the palpebral reflex returned.

Postoperative analgesia was provided with butorphanol 0.5 mg/kg administered intramuscularly approximately 5 cm behind and on the level of the base of the ear 4, 8 and 12 h after the end of anaesthesia. If the pigs showed signs of discomfort or pain later, the administration of butorphanol was prolonged as required. The pigs were offered food 4 to 6 h after extubation. Pre-surgical antibiotic prophylaxis with amoxicillin-clavulanic acid 9 mg/kg was administrated intramuscularly 30 min before the skin incision and then orally once a day for the following 10 days per the requirements of the primary study.

### Experimental design

The surgical site was prepared by clipping of the hair immediately preceding the procedure. After removal of the hair, the skin was scrubbed, dried and prepared aseptically using propan-2-ol and benzalkonium chloride (Cutasept F, Bode, Germany), first with the spray technique and 2 min later with the paint technique. Five minutes later, the surgical site was draped, and a 15-cm-long skin incision was performed on the lateral aspect of the femoral region from the trochanter major to the genual region. Superficial gluteal, biceps femoris and vastus lateralis muscles were separated to expose the sciatic nerve. The fascia was approximated with interrupted sutures using 2–0 lactomer 9–1. The subcutaneous tissue was sutured with a continuous suture using 2–0 lactomer 9–1 (dorsal half of the wound) and 2–0 glycomer 631 (ventral half of the wound), and bites were placed 8 to 10 mm apart. The skin was sutured with a continuous subcuticular suture using 3–0 lactomer 9–1 (dorsal half of the wound) and 3–0 glycomer 631 (ventral half of the wound), with six throws placed on each knot to assure the safety and comparison of the knots at the beginning and end of the continuous suture. All surgical procedures were performed by the same surgeon.

Skin incisions were assessed once daily by a blinded researcher unaware of the use and placement of the suture material. They were evaluated for erythema around the wound (absent, mild = less than 5 mm and pink, moderate = between 5 and 20 mm and red-violet, severe = more than 20 mm and violet), swelling around the wound (absent, mild = less than 5 mm, moderate = between 5 and 20 mm, severe = more than 20 mm), discharge (absent, mild = small amounts of serous or serosanguineous discharge, moderate = large amounts of serous or serosanguineous discharge or mucous discharge, severe = purulent discharge), and dehiscence (absent, mild = less than 10% of the wound, moderate = 10–30% of the wound, severe = more than 30% of the wound), and the parameters were recorded as absent (0), mild (1), moderate (2) or severe (3). The sum of the parameters, called the clinical reactive score (CRS), was calculated on postoperative days 7 and 14. The highest possible CRS was 12. The pigs were also evaluated for signs of discomfort or pain (agitation, vocalization, changes in facial expression, deviation from normal behaviour and appetite, deviation from normal posture and movement) for 12 h after extubation and then three times a day.

Fourteen days after surgery, the pigs were sedated and anaesthetised using the same anaesthesia protocol as that used for the initial surgery, after which they were euthanatised with T − 61 euthanasia solution 0.3 mL/kg intravenously. The skin around the site of the incision was scrubbed, dried and disinfected twice using propan-2-ol and benzalkonium chloride with the spray technique. The contact time of disinfectant was 2 min for the first and 5 min for the second skin preparation. Biopsies of the skin and subcutaneous tissue were taken from the dorsal and ventral part of the wound with 2-cm margins of adjacent skin and associated deeper tissue (to the first muscle plane). Samples were halved, and one half was saved in a sterile collecting pot for immediate microbiological evaluation. The other half was fixed in 10% buffered formalin for histopathologic examination. All examiners were blinded.

### Histopathologic examination

Tissue sections transverse to the incision were taken and routinely embedded in paraffin, sectioned at 4 μm and stained with hematoxylin and eosin. During histopathologic examination, the tissue response to subcuticularly placed suture material was scored by a system modified from that of Sewel et al. [32] and Fick et al. [13]. For the evaluation of the tissue reaction, seven variables were assessed semi-quantitatively and scores multiplied by their weighting factors: the presence of extravascular neutrophils, macrophages, multinucleated giant cells, lymphocytes, fibroblasts and bacterial colonies, and the overall severity of the inflammatory response to the suture material. The absence of a variable was scored as zero, and the presence of a variable was scored from 1 to 3 (presence of bacterial colonies) or 1 to 5 (all other variables) so that score 1 represented the mildest and scores 3 (for presence of bacterial colonies) and 5 (for all other variables) represented the most severe presentation of the variables. In the next step scores were multiplied by their weighting factors (WF). In the scoring system of Sewel et al. [32] and Fick et al. [13], different WF are applied to different variables so that variations in cell types and cell densities are considered in view of the interpretation of the tissue reaction. The WF were as follows: for the presence of extravascular neutrophils (WF − 6), macrophages (WF − 1), multinucleated giant cells (WF − 2), lymphocytes (WF − 2), fibroblasts (WF − 1) and bacterial colonies (WF − 3), and the overall severity of the inflammatory response to the suture material (total estimated cell number) (WF − 3). In the last step, the results of multiplication were summed up to get an aggregate tissue irritation score (ATIS). A more negative ATIS was associated with more severe tissue reaction and poorer wound healing response. The score for the most severe possible ATIS was − 8 (Table 3).

**Table 3:** Variables assessed to calculate the aggregate tissue irritation score (ATIS)

Variable	Score^a^	Weighting factor	Maximum score
Extravascular neutrophils	0–5	− 6	− 30
Macrophages	0–5	− 1	− 5
Multinucleated giant cells	0–5	− 2	− 10
Lymphocytes	0–5	− 2	− 10
Fibroblasts	0–5	− 1	− 5
Overall severity of inflammatory response to suture material (total estimated cell number)	0–5	− 3	− 15
Bacterial colonies	0–3	− 3	− 9
ATIS			− 84

^a^The absence of the variable was scored as zero, and its presence was semi-quantitatively scored in an order increasing from 1 to 3 (presence of bacterial colonies) or 1 to 5 (all other variables)

Suture extrusion, the presence of hyperplastic epidermis and single small pustules were not included in the scoring system of Sewel et al. [32] and Fick et al. [13].

### Microbiological examination

Skin samples and samples of tissue around the suture material were inoculated onto two different solid media: nutrient agar (Oxoid, Hampshire, UK) supplemented with 5% sheep blood for bacteriological examination and Sabouraud dextrose agar (Oxoid, Basingstoke, UK) with chloramphenicol (100 mg/L) for mycological examination. Blood agar plates were prepared in a duplicate for incubation in aerobic and anaerobic atmosphere. Cultures were incubated at 37 °C for up to 4 days, checked for microbial growth and subcultures were performed when necessary. Isolated colonies were further examined. The biochemical characteristics were evaluated using commercial kits API Coryne System (bioMerieux, Marcy I’Etoile, France) and the strain was determined to the species level.

### Statistical analysis

Normal distribution of data was tested by the Shapiro-Wilcox test. The non-parametric Mann-Whitney test was used to compare the two suture materials with respect to the CRS and ATIS, and the results are presented as median (minimum, maximum). Fisher’s exact test was used to compare suture extrusion, the presence of hyperplastic epidermis and single small pustules. Differences were considered significant at a value of *p* < 0.05.

The data were analysed with the Statistical Package for the Social Sciences (IBM SPSS Statistics for Windows, Version 22.0, Armonk, NY: IBM Corp.).

## Data Availability

The data generated or analysed during the current study are available from the corresponding author on reasonable request.

## Abbreviations

ATIS: Aggregate tissue reactive score
CRS: Clinical reactive score
SM: Suture material
